# Factors influencing the difference in dissolved ion inputs to the forest floor between deciduous and coniferous stands: comparison under high and low atmospheric deposition conditions

**DOI:** 10.1007/s10661-023-12132-6

**Published:** 2023-12-02

**Authors:** Naohiro Imamura, Nobuhito Ohte, Nobuaki Tanaka

**Affiliations:** 1 Hokkaido Research Center, Forestry and Forest Products Research Institute, Toyohira-Ku, Sapporo, Hokkaido Japan; 2 Graduate School of Informatics, Kyoto University, Sakyo-Ku, Kyoto, Japan; 3 Graduate School of Agricultural and Life Sciences, The University of Tokyo, Bunkyo-Ku, Tokyo, Japan; 4 The University of Tokyo Hokkaido Forest, The University of Tokyo Forests, Furano, Hokkaido Japan

**Keywords:** Dry deposition, Canopy exchange, Phenology, Atmospheric deposition, Canopy budget model

## Abstract

It is necessary to clear the relationship between physical and vegetation factors on the processes governing dissolved ion inputs to the forest floor to estimate correctly the values of atmospheric input to the forest. This study identified the factors influencing the differences in dissolved ion inputs to the forest floor between coniferous evergreen and broad-leaved deciduous species by analyzing the phenological variations of dry deposition and canopy exchange calculated by the canopy budget model under a high-deposition site near the city of Tokyo and a low-deposition site 84 km further away. At low-deposition site, vegetation factors such as capture efficiency did not explain the differences in Na^+^ or Cl^−^ dry deposition. Leaf physiological characteristics influenced the differences in the Mg^2+^ and Ca^2+^ canopy leaching values, and phenology, leaf wettability, and diffusion processes from water film into leaves influenced the differences in NH_4_^+^ and NO_3_^−^ input processes between tree types. At the high-deposition site, differences in the dry deposition of Na^+^, SO_4_^2−^, Cl^−^, Mg^2+^, Ca^2+^, NH_4_^+^, and NO_3_^−^ between tree types were influenced by differences in capture efficiency between coniferous and broad-leaved canopies in the leafed period and by the absence of leaves in deciduous species after leaf fall. These results indicated that atmospheric deposition affected the capture efficiency of coniferous trees for dry deposition and enhanced the difference of dissolved ion inputs to the forest floor between coniferous and deciduous species.

**Supplementary Information:**

The online version contains supplementary material available at 10.1007/s10661-023-12132-6.

## Introduction

Atmospheric deposition has caused a serious decline in forest health in Europe and Japan (Sase et al., 1998; Schütt & Cowling, 1985; Takamatsu et al., 2001) and the acidification of water and damage to fish and other aquatic life in lakes and rivers in Europe and North America (Baker, 1991; Rodhe, 1972). However, emissions of SO_2_ have decreased due to regulation and controls implemented in the 1970s (Smith et al., 2011), and emissions of NO_x_ have decreased since the 1990s in Europe and since the 2000s in North America and Japan (Akimoto, 2003; EPA, 2022; Kopáček & Posch, 2011). Initial signs of the recovery from forest soil acidification, lake and river acidification, and biotic diversity have been reported in those regions due to the recovery of atmospheric conditions (Eimers et al., 2004; McHale et al., 2017; Sase et al., 2019; Wright et al., 2006). Therefore, correct and wide area estimation for values of atmospheric input to the forest is important to evaluate the recovery potential of the forest by reducing atmospheric deposition.

Stand deposition (SD) to the forest floor represents the total deposition supplied by throughfall (TF) and stemflow (SF). SD can be classified as wet deposition (WD; mainly precipitation), dry deposition (DD; gases and particles during dry periods), and canopy exchange (CE; canopy leaching or uptake) (Parker, 1983). WD is impacted by the strength and proximity of emission sources and meteorological factors, such as precipitation and wind speed. DD is affected by emission sources, meteorological factors, and vegetation factors such as the canopy’s ability to capture pollutants (Andersen & Hovmand, 1999; Lovett et al., 1996). CE encompasses the exchange of gases, such as nitrogen compounds (e.g., NH_3_, NO, and NO_2_), SO_2_, HNO_3_, and dissolved compounds (e.g., SO_4_^2−^, NO_3_^−^, NH_4_^+^). The CE of gases occurs mainly via the stomata of vegetation (Sparks, 2009) and is controlled by stomatal conductance and gas concentrations. The CE of dissolved compounds is controlled by ion concentrations in leaves and precipitation characteristics (Lovett et al., 1996; Parker, 1983). Thus, the input of dissolved ions to the forest floor is influenced by both physical factors (e.g., the strength and proximity of emission sources and meteorological conditions) and vegetation factors (e.g., the canopy form and leaf ion concentrations). Understanding the influence of these physical and vegetation factors on dissolved ion input processes is necessary to clarify the differences in inputs to forests according to composition.

Physical factors can be investigated by comparing deposition trends in stands of the same species among different areas. For example, Imamura et al. (2020a) explained how physical factors (e.g., the distance from the center of Tokyo and elevation) affected the DD process in forests by comparing the WD and DD around Japanese cedar trees (*Cryptomeria japonica* (L.f.) D.Don) at seven sites within the Tokyo Metropolis. By contrast, to clarify vegetation factors, researchers have compared seasonal changes in DD and CE between coniferous and deciduous species at neighboring or nearby locations. Studies have performed seasonal comparisons of deciduous forests dominated by sugar maple (*Acer saccharum* Marsh.) and coniferous forests dominated by white pine (*Pinus strobus* L.) in Ontario, Canada (Neary & Gizyn, 1994); deciduous forests dominated by sugar maple and coniferous forests dominated by balsam fir (*Abies balsamea* (L.) Mill.) in Quebec, Canada (Houle et al., 1999); and deciduous forests dominated by silver birch (*Betula pendula* Roth) and coniferous forests dominated by Corsican pine (*Pinus nigra* ssp. *laricio* Maire) in Merksplas, Belgium (De Schrijver et al., 2004; Table 1). From these previous studies, the influence of vegetation factors (e.g., leaf capture efficiency, physiological activity, and leafless period in deciduous species) on the processes governing dissolved element inputs to the forest floor could be investigated by comparing seasonality between coniferous and deciduous species at neighboring locations. However, reported vegetation factors differed among previous studies.

**Table 1:** Comparison of deposition values and study site characteristics between this and previous studies

Site locations		Ontario, Canada	Quebec, Canada	Merksplas, Belgium	Chichibu, Japan	Tanashi, Japan
Wet deposition (mmol m^–2^ year^–1^)	Na^+^	4.1	2.2	132.0	10.1	36.8
	Cl^−^	5.6	3.1	-	11.8	47.2
	SO_4_^2−^	30.5	23.6	-	9.4	19.5
	K^+^	3.0	0.9	9.6	11.3	10.5
	Mg^2+^	2.1	0.7	19.2	2.5	8.0
	Ca^2+^	8.1	2.8	38.4	8.3	17.6
	H^+^	62.8	55.9	6.0	8.5	25.8
	NH_4_^+^	25.8	23.4	143.5	13.6	27.6
	NO_3_^−^	38.3	30.4	111.6	16.3	36.2
Tree species	Deciduous	*Acer saccharum*, *Acer rubrum*, *Fagus grandifolia*, *Betula alleghaniensis*, *Populus grandidentata* *Betula papyrifera*	*Acer saccharum*	*Betula pendula*	*Fagus crenata* *Fagus japonica* *Tsuga sieboldii*	*Quercus acutissima* *Pinus densiflora* *Quercus rubra*
	Coniferous	*Pinus strobus*, *Tsuga canadensis*, *Acer rubrum*, *Quercus rubra*, *Betula papyrifera*	*Abies balsamea*, *Picea rubens*	*Pinus nigra*	*Cryptomeria japonica*	*Cryptomeria japonica*
Tree age	Deciduous		80–120	40	210–250^‡^	80
	Coniferous	87*	50	40	47–57	51
Tree height (m)	Deciduous		20.2	14.4	24.5	18.5
	Coniferous		12.6	16.1	30.5	16.3
Basal area (m^2^ ha^−1^)	Deciduous	23.5^†^	24.3	16	33.2 (> 5 cm)^‡^	25.6 (> 5 cm)
	Coniferous	31.3^†^	32.6 (> 9 cm)	45	88.6	40.0 (> 5 cm)
LAI (m^2^ m^−2^)	Deciduous		5.5	Three times higher at Evergreen site than Deciduous site	2.86	1.92
	Coniferous		5		1.81	1.92
Reference		Neary and Gizyn (1994)	Houle et al. (1999)	De Schrijver et al. (2004)	This study	This study

^*^Watmough, S.A., & Dillon, P.J. (2004). Major element fluxes from a coniferous catchment in central Ontario, 1983–1999. *Biogeochemistry*. *67*, 369–398

^†^Watmough, S.A., & Dillon, P.J. (2003). Base cation and nitrogen budgets for seven forested catchments in central Ontario, 1983–1999. *Forest Ecology and Management*. *177*, 155–177

^‡^Sawada, H., Kaji, M., Oomura, K., & Ohkubo, T. (2008). Age structure and regeneration characteristic in a natural beech (*Fagus japonica* Maxim. And *F. crenata* Blume) forest in the Chichibu mountains, central Japan. *Bulletin of the Tokyo University Forests*, *119*, 1–23

De Schrijver et al. (2007) summarized the differences in annual SD between coniferous and deciduous stands reported in 38 case studies of sites with different atmospheric conditions. They found that, among stands, the Na^+^ SD increased with open field deposition; there were no relationships of the variations in the SDs of K^+^, Ca^2+^, and Mg^2+^ with open field deposition; moreover, the NH_4_^+^ SD had a stronger correlation with open field deposition than did the NO_3_^−^ SD (De Schrijver et al., 2007). From these results, we hypothesized that open field deposition, which is driven by physical factors, also affects the differences in DD and CE between coniferous and deciduous species, which are influenced by vegetation factors. The difference in atmospheric deposition could affect the different results about vegetation factors among previous studies. The present study aimed to identify factors influencing the differences in annual SDs between coniferous evergreen and broad-leaved deciduous species by comparing DD and CE between species during different phenological phases at sites that experience high and low atmospheric deposition values.

## Materials and methods

### Site description

**Fig. 1:**
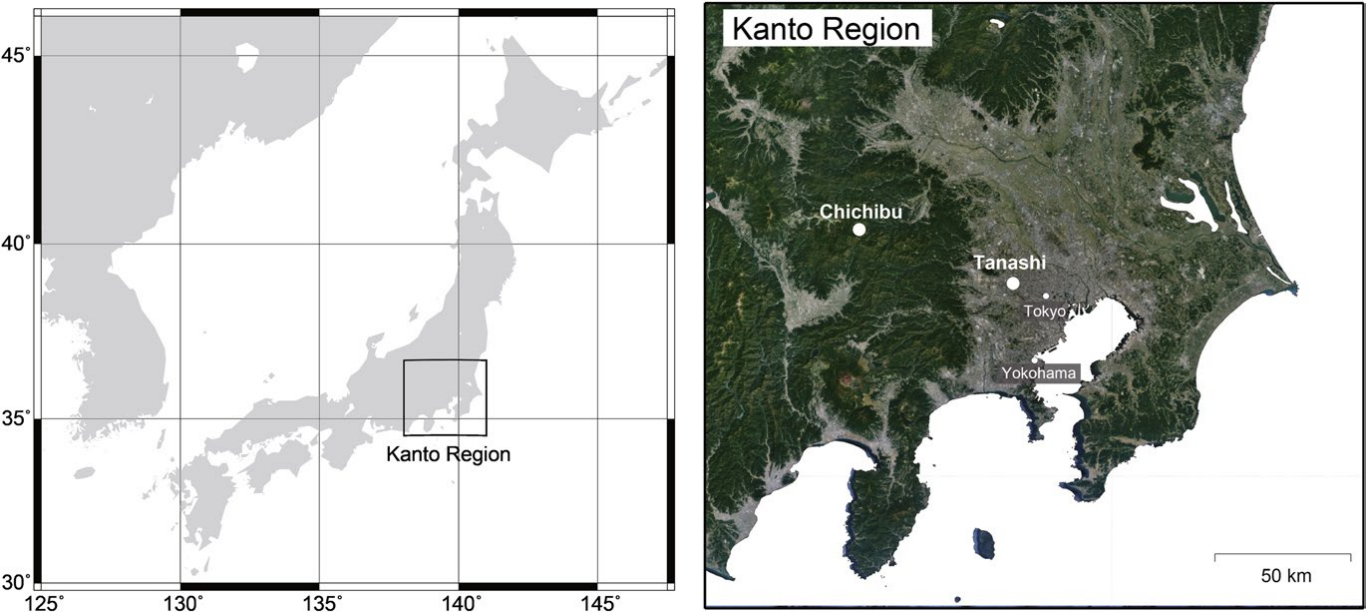
Location of the Chichibu and Tanashi forest sites. The satellite image was obtained from Google Maps

We established forest sites in Kanto, Japan, experiencing either lower atmospheric deposition (Chichibu) or higher atmospheric deposition (Tanashi) (Fig. 1 and Table 1). The Chichibu site was located at The University of Tokyo Chichibu Forest, 83.7 km from the center of Tokyo (Imamura et al., 2020a) and about 100 km from Tokyo Bay. The site hosts a natural deciduous mixed forest (35°56′N, 138°48′E; 1264 m a.s.l.) and coniferous Japanese cedar (*C. japonica*) plantation (35°56′N, 138°49′E; 1040 m a.s.l.) (Fig. 1) (Imamura et al., 2020b). The deciduous mixed forest is dominated by Japanese beech (*Fagus japonica* Maxim.), Siebold’s beech (*Fagus crenata* Blume), and Hemlock fir (*Tsuga sieboldii* Carrière). The Tanashi site was established in The University of Tokyo Tanashi Forest (35°44′N, 139°32′E) (Fig. 1) (Shi et al., 2014). The site is 14.3 km from the center of Tokyo and 22.6 km from Tokyo Bay (Imamura et al., 2020a). The present study focused on naturally seriated secondary deciduous stands of Sawtooth oak (*Quercus acutissima* Carruth.) and Japanese cedar (*C. japonica*) evergreen plantations at the Tanashi site.

### Precipitation, throughfall, and stemflow measurements

**Fig. 2:**
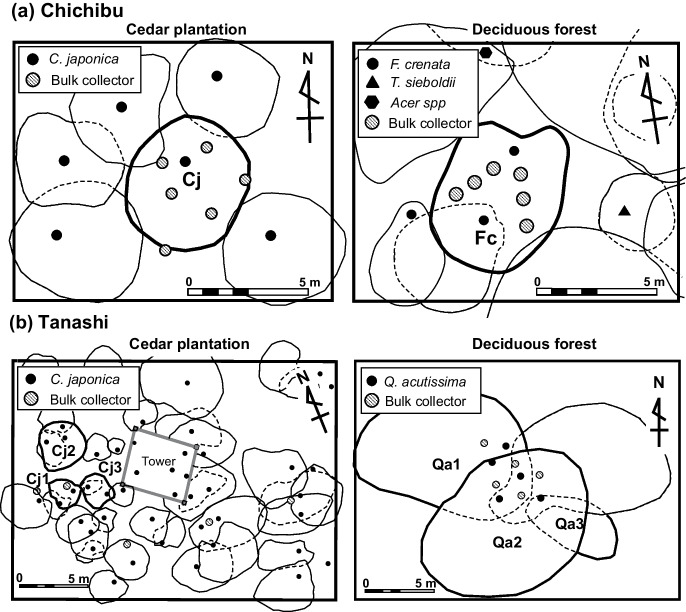
Canopy projection and locations of bulk throughfall samplers at the observation sites at (**a**) Chichibu and (**b**) Tanashi. Individual *Fagus crenata*, *Quercus acutissima*, and *Cryptomeria japonica* trees are denoted as Fc, Qa, and Cj, respectively. Stemflow water was collected from Cj1, Cj2, and Cj3 and from Qa1, Qa2, and Qa3, respectively, at Tanashi

Wet-only precipitation was collected using an automatic wet-only sampler at the First Nursery meteorological station at Tanashi (Imamura et al., 2018). Bulk precipitation was collected using a bulk sampler at the Koakasawa meteorological station at Chichibu and at the meteorological station at Tanashi. At Chichibu, six throughfall collectors arranged in two parallel lines of three samplers each were installed 1 m from broad-leaved deciduous species (*F. crenata*) (Fig. 2) (Imamura et al., 2012). Under the stand of coniferous evergreen species (*C. japonica*), throughfall samplers were placed at random, with six per site. Water samples were collected from the same three collectors to take into account the distance from the trunks (Fig. 2). SF was collected at each study plot. At Tanashi, under a broad-leaved deciduous (*Q. acutissima*) tree and *C. japonica* tree, TF samples were collected using five bulk samplers (Fig. 2). *F. crenata* and *Q. acutissima* belong to Fagaceae (the Beech family). Water samples were collected from all collectors. SF collectors were installed in three individuals of each tree type. Bulk precipitation, TF, and SF were measured and sampled at least once a month from October 2010 to September 2012 at Chichibu and Tanashi. Stemflow volumes were calculated by dividing the volume of collected water by the canopy projection area at each site. Table 1 summarizes the study plot characteristics, including tree age, tree height, basal area, and leaf area index (LAI).

The pH and electrical conductivity of the samples were measured during each sampling period. The concentrations of dissolved inorganic ions (Na^+^, Cl^−^, SO_4_^2−^, K^+^, Mg^2+^, Ca^2+^, NH_4_^+^, and NO_3_^−^) were measured using ion chromatography (LC-10A; Shimadzu Corp., Kyoto, Japan) and flame emission spectrometry (Z-2310; Hitachi High-Tech Science Corp., Tokyo, Japan). For quality control, each sample was assessed by comparing the measured and calculated conductivities of the water samples and the ion balances.

### Meteorological and air quality measurements

Meteorological observations of air temperature and relative humidity were conducted atop a 23-m-high meteorological tower at the deciduous forest site in Chichibu and a 26-m-high meteorological tower at the *C. japonica* plantation in Tanashi. Air temperature and relative humidity were measured using a thermo-hygrograph at Chichibu (CS500; Campbell Scientific, Inc., Logan, USA) and Tanashi (Hobo U23-002; Onset Computer Corp., Bourne, MA, USA).

NO_2_ and NO_x_ (NO plus NO_2_) filter samples were collected monthly from July 26, 2011, to July 5, 2012, on the meteorological tower in Chichibu using a passive sampler (OG-SN-S; Ogawa & Co., Ltd, Kobe, Japan). NO was calculated by subtraction. Water extracted from the exposed filter and blank filter was analyzed using the Saltzman method with a spectrophotometer (U-1800; Hitachi High-Tech Corporation, Tokyo, Japan). The limit of quantitation (LOQ) was calculated as ten times the standard deviation of the blanks (MacDougall et al., 1980). At Tanashi, these concentrations were obtained from national monitoring data obtained from July 14, 2011, to July 3, 2012, at the nearest monitoring site (located 1.2 km from Tanashi) (National Institute for Environmental Studies, 2022).

### Estimation of dry deposition and canopy exchange

The TF and SF volumes were averaged across all collector samples at each site. The volume-weighted mean ion concentrations in TF and SF were then calculated. TF and SF depositions (mmol m^−2^) were calculated using the above datasets. During periods without observations, WD was calculated using bulk deposition and the bulk:wet-only concentration ratio at Tanashi (Imamura et al., 2018). Bulk deposition was used as a proxy for WD at Chichibu during all sampling periods. DD and CE were calculated with the canopy budget model (CBM) using WD, TF, and SF (Adriaenssens et al., 2013; Staelens et al., 2008). The DDs of K^+^, Mg^2+^, and Ca^2+^ were estimated assuming that aerosols containing K^+^, Mg^2+^, and Ca^2+^ were deposited onto the forest canopy at a rate equal to that of particulate Na^+^. Using the net throughfall (NTF):WD ratio of the Na^+^ tracer ion, the DD rates of K^+^, Mg^2+^, and Ca^2+^ were calculated as follows:

2.1 $${DD}_{X}=\frac{{(TF+SF-WD)}_{Na}}{{WD}_{Na}}\bullet {WD}_{X}$$

where _*X*_ is K^+^, Mg^2+^, or Ca^2+^.

The CL values of K^+^, Mg^2+^, and Ca^2+^ were calculated by subtracting DD from NTF. The CL of basic cations (BC; K^+^  + Mg^2+^  + Ca^2+^) should equal the CU of H^+^ (Cronan & Reiners, 1983) and NH_4_^+^ (Roelofs et al., 1985) based on the ion charge balance of the canopy (Eq. 2.2):

2.2 $${CU}_{{NH}_{4}+H}={CL}_{BC}$$

H^+^ has an exchange capacity six times larger than that of NH_4_^+^ (Draaijers et al., 1998), which is accounted for by the relative uptake efficiency factor (*xH* = 6) in Eq. 2.3 to calculate the CU of NH_4_^+^ (De Schrijver et al., 2004):

2.3 $${CU}_{{NH}_{4}}=\frac{{(PD+TF+SF)}_{{NH}_{4}}}{{(PD+TF+SF)}_{{NH}_{4}}+xH\bullet {(PD+TF+SF)}_{H}}\bullet {CL}_{BC}$$

The CU of H^+^ was calculated by subtracting NH_4_ from CU_NH4+H_. The DDs of NH_4_^+^ and H^+^ were calculated by subtracting CU from NTF.

The CU of NO_3_^−^ was calculated based on the TF fluxes of NH_4_^+^ and NO_3_^−^, using an efficiency factor of NH_4_^+^ versus NO_3_^−^ uptake (xNH_4_) with a value of six (de Vries et al., 2001; Eq. 2.4).

2.4 $${CU}_{({NO}_{3}+{NH}_{4})}=\frac{{xNH}_{4}\bullet {\left(TF+SF\right)}_{{NH}_{4}}+{(TF+SF)}_{{NO}_{3}}}{{xNH}_{4}\bullet {(TF+SF)}_{{NH}_{4}}}\bullet {CU}_{{NH}_{4}}$$

Na^+^, Cl^−^, and SO_4_^2−^ were defined only in terms of DD, i.e., not in terms of CL or CU.

### Definition of seasons

**Fig. 3:**
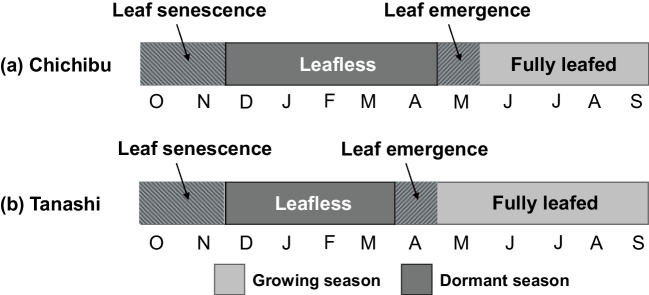
Phenological periods at (**a**) Chichibu and (**b**) Tanashi. The leaf emergence period lasted 1 month (May at Chichibu and April at Tanashi). The fully leafed period was 4 months (June–September) at Chichibu and 5 months (May–September) at Tanashi. The leaf senescence period was 2 months (October–November) at both sites. The leafless period was 5 months (December–April) at Chichibu and 4 months (December–March) at Tanashi

At Chichibu, long-term video data indicate that the leaf-out period for the *F. crenata* canopy lasts from May to November (Fujiwara & Saito, 2005); these 7 months were considered the growing season, and the remaining 5 months were considered the dormant season (Fig. 3). At Tanashi, the leaf-out period for *Q. acutissima* lasted from April until November based on visual observations; these 8 months were defined as the growing season, and the remaining 4 months were considered the dormant season (Fig. 3).

The 2-year average annual DD and CE values were separated into four phenological phases (Staelens et al., 2007; Van Stan et al., 2012). The leaf emergence and leaf senescence periods were defined as the first and last 2 months of the growing season, respectively, for all species (Fig. 3). The other months of the growing season were defined as the fully leafed period. The leafless period was equal to the duration of the dormant season (Fig. 3).

### Data analysis

To elucidate the differences in annual SDs between coniferous and deciduous species, annual SDs between coniferous and deciduous stands were compared at original study sites (Chichibu and Tanashi) and previous study sites (Ontario; Neary & Gizyn, 1994, Quebec; Houle et al., 1999, Merksplas; De Schrijver et al., 2004). Previous studies have performed seasonal comparisons of deciduous and coniferous forests. A low atmospheric deposition site was defined as a lower wet deposition site compared to that at Chichibu. By contrast, a higher atmospheric deposition site was defined as a higher wet deposition site compared to that at Tanashi. DD and CE in annual, growing, and dormant seasons were calculated by the CBM at Chichibu, Tanashi, Ontario, Quebec, and Merksplas. In addition, only at Chichibu and Tanashi, monthly averaged DD and CE were calculated by the same method in the leaf emergence, fully leafed, leaf senescence, and leafless periods. A paired *t*-test was used to test the significant difference in annual and seasonal SD, DD, and CE between coniferous and deciduous stands.

## Results

### Meteorology and air quality

**Fig. 4:**
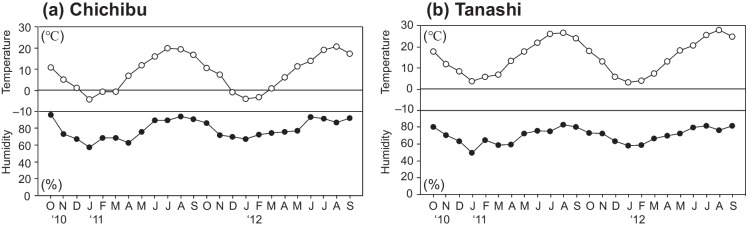
Monthly air temperature (°C) and relative humidity (%) by canopy height from October 2010 to September 2012 at (**a**) Chichibu and (**b**) Tanashi

Figure 4 presents the seasonal variations in meteorological parameters of the canopies at Chichibu and Tanashi from October 2010 to September 2012. The annual mean humidity was 79% and 70% at Chichibu and Tanashi, respectively. The relative humidity was higher at Chichibu than Tanashi throughout the year, particularly in summer when the relative humidity at Chichibu exceeded 80% from June to September.

At Chichibu, the average absorption of the field blank for NO_2_ and NO_x_ was 0.16 (*n* = 4) and 0.21 (*n* = 4), respectively. The LOQ of absorption was 0.02 and 0.57 for NO_2_ and NO_x_. Whereas all values of absorption for NO_2_ were above the LOQ, those for NO_x_ were below LOQ. Therefore, the average NO and NO_2_ concentrations were below the LOQ and 1.2 ppb, respectively, at Chichibu. At Tanashi, the average NO and NO_2_ concentrations were 5.5 ppb and 16.6 ppb, respectively. The NO_2_ concentrations were > 10 times higher at Tanashi than Chichibu.

### Differences in stand deposition between deciduous and coniferous species

**Fig. 5:**
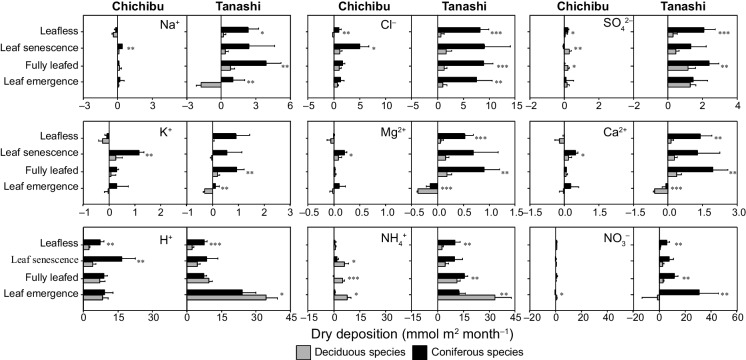
The 2-year average dry deposition values (mmol m^−2^ month^−1^) of Na^+^, Cl^−^, SO_4_^2−^, K^+^, Mg^2+^, Ca^2+^, H^+^, NH_4_^+^, and NO_3_^−^ during each canopy phenological phase for deciduous species and coniferous species (*Cryptomeria japonica*) at Chichibu and Tanashi. The leaf emergence, fully leafed, leaf senescence, and leafless periods are defined in Fig. 3. *A paired *t*-test, *p* < 0.05, **t-test, *p* < 0.01, ***t-test, *p* < 0.001

At Chichibu, there were no differences in the 2-year average annual SD and DD values for Na^+^ (*p* = 0.05) and SO_4_^2−^ (*p* = 0.61) between deciduous and coniferous species (Tables 2 and 3). By contrast, the average annual SD and DD values of Cl^−^ were significantly higher for coniferous than deciduous species (*p* < 0.05) (Tables 2 and 3). At Tanashi, the average annual SD and DD values of Na^+^, Cl^−^, and SO_4_^2−^ were significantly higher for coniferous than deciduous species (*p* < 0.001) (Tables 2 and 3). At Chichibu, the DD values were significantly higher for coniferous than deciduous species for Na^+^ and Cl^−^ in the leaf senescence period (*p* < 0.05) and Cl^−^ and SO_4_^2−^ in the leafless period (*p* < 0.05) (Fig. 5). The SO_4_^2−^ DD value was significantly higher for deciduous than coniferous species in the fully leafed and leaf senescence periods (*p* < 0.05) (Fig. 5). At Tanashi, the DD values were significantly higher for coniferous than deciduous species during all phenological periods except the leaf senescence period for Na^+^ (*p* < 0.05) and Cl^−^ (*p* < 0.01) and the fully leafed and leafless periods for SO_4_^2−^ (*p* < 0.01) (Fig. 5).

**Table 2:** Comparison of the average annual water and ion throughfall deposition (TF), stemflow deposition (SF), and stand deposition (SD) values between deciduous (Dec) and coniferous (Con) trees in this and previous studies

		Flux (mmol m^–2^ year^–1^)						Reference
Flux		TF		SF		SD		
Species		Dec	Con	Dec	Con	Dec	Con	
Ontario	Water	929.0	819.3	32.0	20.0	950.3 (145.8)	832.7 (82.4)	Neary and Gizyn (1994)
	Na^+^	4.2	4.8	0.2	0.1	4.3 (1.2)	4.9 (0.4)	
	Cl^–^	8.6	9.2	0.6	0.4	9.0 (1.4)	9.5 (1.5)	
	SO_4_^2–^	34.2	42.8	3.5	3.6	36.5 (0.9)	45.2 (4.6)	
	K^+^	27.7	25.3	5.2	2.2	31.1 (8.4)	26.8 (2.3)	
	Mg^2+^	6.8	7.2	0.7	0.4	7.3 (0.4)	7.4 (0.6)	
	Ca^2+^	18.8	23.3	2.3	1.9	20.4 (0.6)	24.6 (2.6)	
	H^+^	42.3	61.3	0.9	3.2	42.9 (7.9)	63.4 (8.8)	
	NH_4_^+^	19.0	15.0	0.3	0.1	19.2 (3.6)	15.0 (2.7)	
	NO_3_^–^	39.3	45.3	1.3	0.5	40.2 (3.8)	45.6 (7.3)	
Quebec	Water	1165.0	1074.0	-	-	1165.0 (94.0)	1074.0 (100.0)	Houle et al. (1999)
	Na^+^	3.5	3.7	-	-	3.5 (1.1)	3.7 (1.0)	
	Cl^−^	6.8	8.6	-	-	6.8 (1.4)	8.6 (1.7)	
	SO_4_^2−^	28.2	33.8	-	-	28.2 (3.6)	33.8 (5.7)	
	K^+^	41.0	27.7	-	-	41.0(10.1)	27.7 (5.4)	
	Mg^2+^	4.4	5.3	-	-	4.4 (0.5)	5.3 (0.7)	
	Ca^2+^	12.6	13.6	-	-	12.6 (1.1)	13.6 (2.0)	
	H^+^	34.7	46.7	-	-	34.7 (7.9)	46.7 (9.8)	
	NH_4_^+^	18.2	9.8	-	-	18.2 (2.9)	9.8 (1.9)	
	NO_3_^−^	33.8	25.8	-	-	33.8 (2.9)	25.8 (2.4)	
Merksplas	Water	-	-	-	-	-	-	De Schrijver et al. (2004)
	Na^+^	158.4	202.8	-	-	158.4 (121.2)	202.8 (163.2)	
	Cl^−^	-	-	-	-	-	-	
	SO_4_^2−^	-	-	-	-	-	-	
	K^+^	44.4	36.0	-	-	44.4 (45.6)	36.0 (28.8)	
	Mg^2+^	14.4	18.0	-	-	14.4 (12.0)	18.0 (14.4)	
	Ca^2+^	22.8	26.4	-	-	22.8 (14.4)	26.4 (20.4)	
	H^+^	4.8	0.0	-	-	4.8 (4.8)	0.0 (1.2)	
	NH_4_^+^	194.4	569.3	-	-	194.4(125.0)	569.3(371.8)	
	NO_3_^−^	170.1	356.1	-	-	170.1 (95.7)	356.1(196.6)	
Chichibu	Water	1422.2	1162.2	79.5	107.4	1501.7 (100.5)	1269.6 (58.8)	This study
	Na^+^	8.6	9.4	0.5	1.1	9.0 (1.3)	10.6 (0.9)	
	Cl^–^	16.5	28.6	1.0	5.9	17.5 (2.7)	34.5 (7.1)	
	SO_4_^2–^	10.4	9.2	0.8	1.1	11.3 (1.3)	10.4 (0.4)	
	K^+^	83.1	66.7	6.7	7.3	89.8 (26.6)	74.0 (12.3)	
	Mg^2+^	6.0	8.6	0.6	0.5	6.6 (0.6)	9.1 (1.1)	
	Ca^2+^	14.6	35.7	1.4	2.6	16.1 (2.2)	38.3 (2.8)	
	H^+^	1.7	2.5	0.1	8.9	1.8 (0.2)	11.4 (1.7)	
	NH_4_^+^	17.8	3.9	1.3	0.6	19.1 (1.6)	4.5 (2.2)	
	NO_3_^–^	16.5	13.2	0.3	0.1	16.8 (1.7)	13.3 (1.4)	
Tanashi	Water	1331.4	1328.8	22.7	134.1	1354.1 (86.8)	1462.8 (164.8)	This study
	Na^+^	39.4	62.0	1.0	9.5	40.4 (5.0)	71.5 (8.5)	
	Cl^−^	58.9	120.1	1.7	29.9	60.6 (8.1)	150.0 (16.3)	
	SO_4_^2−^	28.1	36.0	0.6	7.9	28.7 (3.2)	43.9 (4.2)	
	K^+^	119.3	79.1	6.1	17.7	125.4 (9.1)	96.8 (6.5)	
	Mg^2+^	20.4	24.7	0.4	3.1	20.7 (2.0)	27.8 (5.1)	
	Ca^2+^	39.5	57.7	0.8	5.5	40.2 (4.1)	63.1 (10.7)	
	H^+^	9.6	4.6	0.6	23.1	10.2 (3.2)	27.7 (1.6)	
	NH_4_^+^	72.9	112.1	0.7	16.7	73.5 (9.5)	128.8 (19.4)	
	NO_3_^−^	50.7	110.0	1.0	24.0	51.7 (7.7)	134.0 (19.7)	

Values in parentheses are standard deviations

**Table 3:** Comparison of the dry deposition values (mmol m^−2^ year^−1^) of Na^+^, Cl^−^, SO_4_^2−^, K^+^, Mg^2+^, Ca^2+^, H^+^, NH_4_^+^, and NO_3_^−^ between deciduous (Dec) and coniferous (Con) species during the annual, growing, and dormant periods in this and previous studies

Dry deposition (mmol m^–2^ year^–1^)							
Elements	Annual		Growing		Dormant		Site
	Dec	Con	Dec	Con	Dec	Con	
Na^+^	0.4 (0.6)	0.7 (0.2)	− 0.2 (0.2)	− 0.3 (0.2)	0.6 (0.6)	0.9 (0.1)	Ontario
	1.3	1.6	0.6	0.5	0.8	1.0	Quebec
	26.4	70.8	14.0	33.6	12.5	31.0	Merksplas
	− 1.0 (1.3)	0.5 (0.8)	0.8 (1.8)	1.4 (0.4)	− 1.8 (1.3)	− 0.9 (0.5)	Chichibu
	3.6 (5.6)	34.7 (8.5)	2.9 (3.5)	25.3 (6.0)	0.8 (2.3)	9.4 (3.7)	Tanashi
Cl^−^	3.7 (0.8)	3.7 (0.7)	2.9 (0.5)	1.8 (0.2)	0.9 (0.3)	1.7 (0.9)	Ontario
	3.7	5.4	2.5	3.4	1.2	2.1	Quebec
	-	-	-	-	-	-	Merksplas
	5.7 (2.7)	22.7 (7.0)	6.8 (5.1)	17.9 (5.7)	− 1.1 (1.4)	4.8 (2.1)	Chichibu
	13.4 (9.0)	102.8 (16.3)	10.7 (5.3)	70.0 (11.5)	2.8 (3.9)	32.8 (7.0)	Tanashi
SO_4_^2−^	5.0 (4.4)	15.7 (4.9)	3.0 (1.3)	5.9 (0.6)	2.3 (3.5)	9.9 (5.5)	Ontario
	4.6	10.1	2.7	3.4	1.9	6.7	Quebec
	-	-	-	-	-	-	Merksplas
	1.8 (1.2)	0.9 (0.7)	1.6 (1.2)	− 0.1 (0.6)	0.3 (1.1)	1.0 (0.5)	Chichibu
	9.2 (3.5)	24.3 (4.2)	8.0 (2.2)	16.0 (2.9)	1.2 (1.6)	8.3 (2.5)	Tanashi
K^+^	0.3 (0.5)	0.4 (0.1)	− 0.3 (0.2)	− 0.3 (0.3)	0.2 (0.1)	0.3 (0.0)	Ontario
	0.5	0.6	0.3	0.2	0.3	0.3	Quebec
	1.9	5.1	1.4	3.3	0.5	1.1	Merksplas
	− 1.5 (1.4)	0.4 (0.9)	0.5 (2.4)	2.7 (0.6)	− 1.2 (1.2)	− 0.4 (0.4)	Chichibu
	0.4 (1.7)	8.5 (2.4)	0.4 (0.9)	5.7 (1.5)	0.0 (1.2)	3.6 (2.1)	Tanashi
Mg^2+^	0.2(0.3)	0.3 (0.1)	− 0.2 (0.1)	− 0.2 (0.2)	0.2 (0.1)	0.3 (0.0)	Ontario
	0.4	0.5	0.3	0.2	0.1	0.2	Quebec
	1.9	5.1	0.9	2.1	1.0	2.6	Merksplas
	− 0.2 (0.3)	0.2 (0.2)	0.2 (0.4)	0.4 (0.1)	− 0.3 (0.4)	− 0.1 (0.1)	Chichibu
	0.9 (1.2)	7.8 (1.9)	0.6 (0.8)	5.5 (1.3)	0.2 (0.4)	2.1 (0.7)	Tanashi
Ca^2+^	0.9 (1.6)	1.3 (0.6)	− 0.5 (0.5)	− 0.6 (0.6)	0.8 (0.7)	1.1 (0.2)	Ontario
	1.7	2.0	1.0	0.9	0.6	0.8	Quebec
	3.8	10.3	1.9	4.5	2.1	5.2	Merksplas
	− 0.4 (1.1)	0.6 (0.7)	0.6 (1.4)	1.3 (0.4)	− 1.0 (1.4)	0.0 (0.4)	Chichibu
	1.8 (2.7)	16.9 (4.1)	1.2 (1.6)	11.4 (2.7)	0.5 (1.2)	5.6 (2.0)	Tanashi
H^+^	33.2 (17.8)	64.5 (18.7)	24.8 (6.9)	35.0 (1.0)	9.4 (9.3)	31.1 (18.8)	Ontario
	36.7	40.2	28.2	22.6	7.7	18.1	Quebec
	4.9	− 5.0	2.4	− 1.3	− 0.7	− 2.5	Merksplas
	62.0 (16.5)	119.2 (20.2)	47.9 (17.5)	79.3 (14.5)	12.5 (6.0)	36.9 (8.3)	Chichibu
	105.8 (15.2)	108.6 (14.9)	96.7 (14.1)	77.6 (12.3)	9.2 (3.9)	30.5 (5.2)	Tanashi
NH_4_^+^	− 2.8 (5.4)	− 7.2 (1.9)	− 0.1 (4.5)	− 3.7 (0.7)	− 1.7 (1.9)	− 2.5 (2.5)	Ontario
	− 0.8	− 11.0	− 1.2	− 10.0	0.9	− 0.8	Quebec
	82.9	446.3	57.1	301.9	26.7	148.2	Merksplas
	41.8 (7.3)	8.6 (5.0)	37.5 (15.5)	5.1 (3.9)	5.1 (3.3)	3.1 (2.6)	Chichibu
	104.2 (16.2)	153.6 (23.0)	94.2 (14.6)	112.3 (14.0)	10.0 (5.9)	40.9 (11.6)	Tanashi
NO_3_^−^	2.2 (2.9)	10.0 (6.8)	− 0.1 (0.6)	3.2 (0.9)	2.3 (2.9)	7.2 (7.2)	Ontario
	4.7	− 3.5	− 0.4	− 6.2	5.1	2.8	Quebec
	63.1	246.6	45.9	166.3	26.7	82.4	Merksplas
	5.9 (1.9)	6.6 (0.6)	1.8 (2.0)	2.6 (1.4)	3.6 (2.2)	3.6 (1.1)	Chichibu
	22.3 (9.3)	107.1 (20.9)	19.0 (6.7)	83.0 (16.7)	3.2 (3.7)	24.0 (8.2)	Tanashi

Values in parentheses are standard deviations

**Fig. 6:**
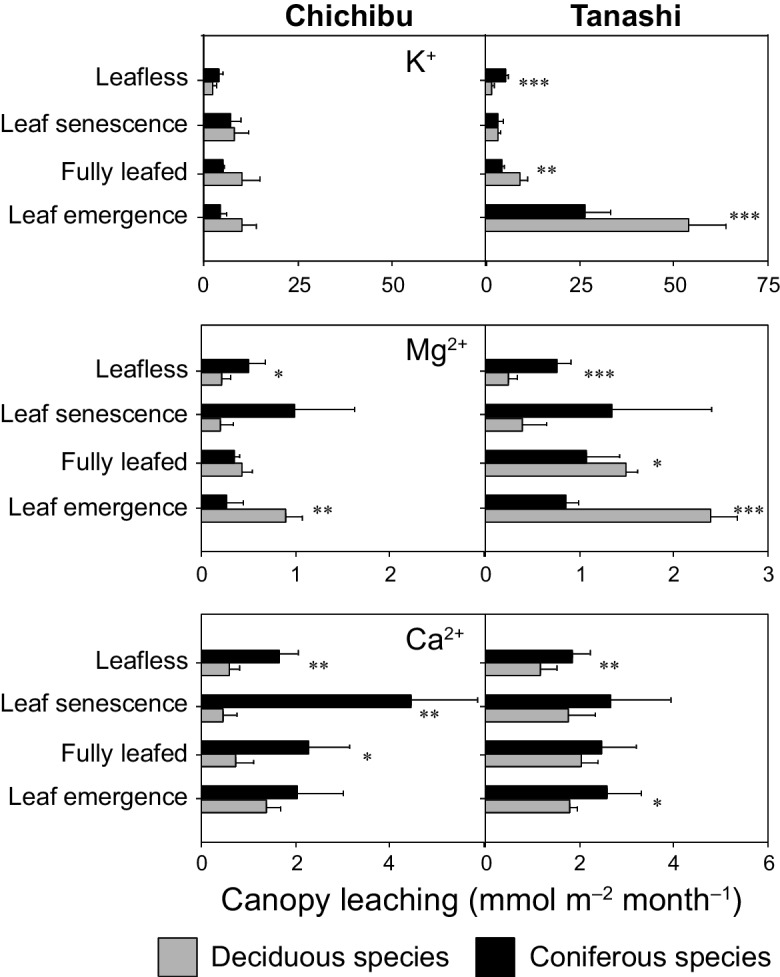
The 2-year average canopy leaching values (mmol m^−2^ month^−1^) of K^+^, Mg^2+^ and Ca^2+^ during each canopy phenological phase for deciduous species and coniferous species (*Cryptomeria japonica*) at Chichibu and Tanashi. The leaf emergence, fully leafed, leaf senescence, and leafless periods are defined in Fig. 3. * A paired *t*-test, *p* < 0.05, **t-test, *p* < 0.01, ***t-test, *p* < 0.001

The annual K^+^ SD value was non-significantly higher for deciduous than coniferous species at Chichibu (*p* = 0.50) and significantly at Tanashi (*p* < 0.01) (Table 2). The annual Mg^2+^ SD value did not differ between deciduous and coniferous species at Chichibu (*p* = 0.09) but was significantly higher for coniferous than deciduous species at Tanashi (*p* < 0.01) (Table 2). The annual SD value of Ca^2+^ was significantly higher for coniferous than deciduous species at both Chichibu and Tanashi (*p* < 0.01) (Table 2). At Chichibu, the annual CL values of K^+^, Mg^2+^, and Ca^2+^ were > 20 times higher than the DD values for both coniferous and deciduous species (Tables 3 and 4). At Tanashi, the annual K^+^ DD value was about one-tenth the CL value for coniferous species, and the Mg^2+^ and Ca^2+^ DD values were about half the respective CL values (Tables 3 and 4). The Mg^2+^ CL value was significantly higher for deciduous than coniferous species in the leaf emergence period at Chichibu (*p* < 0.01) (Fig. 6). At Tanashi, the CL values of K^+^ (*p* < 0.01) and Mg^2+^ (*p* < 0.05) were significantly higher for deciduous than coniferous species in the leaf emergence and fully leafed periods (Fig. 6). By contrast, the Mg^2+^ CL value was significantly higher for coniferous than deciduous species in the leafless period at Chichibu (*p* < 0.05) and Tanashi (*p* < 0.001) (Fig. 6). The Ca^2+^ CL value was significantly higher for coniferous than deciduous species in the fully leafed, leaf senescence, and leafless periods at Chichibu (*p* < 0.05) and in the leaf emergence and leafless periods at Tanashi (*p* < 0.05) (Fig. 6). Coniferous species showed significantly higher DD values of Mg^2+^ (*p* < 0.01) and Ca^2+^ (*p* < 0.01) than deciduous species during all phenological periods except the leaf senescence period at Tanashi (Fig. 5).

**Table 4:** Comparison of the canopy leaching values (mmol m^−2^ year^−1^) of K^+^, Mg^2+^, and Ca^2+^ between deciduous (Dec) and coniferous (Con) species during the annual, growing, and dormant periods in this and previous studies

Canopy leaching (mmol m^–2^ year^–1^)							
Elements	Annual		Growing		Dormant		Site
	Dec	Con	Dec	Con	Dec	Con	
K^+^	27.8 (8.6)	23.5 (1.9)	22.8 (5.6)	18.9 (1.5)	5.2 (2.8)	5.0 (1.2)	Ontario
	39.6	26.2	37.0	20.8	2.1	5.4	Quebec
	32.9	21.3	28.0	14.2	4.5	7.9	Merksplas
	80.0 (25.3)	62.4 (12.2)	67.7 (31.2)	40.1 (8.0)	11.5 (6.3)	20.3 (5.2)	Chichibu
	114.4 (10.1)	77.8 (7.6)	107.3 (10.5)	55.5 (6.4)	7.1 (2.7)	21.5 (4.3)	Tanashi
Mg^2+^	5.0 (0.9)	5.0 (0.4)	4.1 (0.2)	3.4 (0.3)	1.0 (0.6)	1.9 (0.6)	Ontario
	3.2	4.0	2.9	2.6	0.4	1.5	Quebec
	2.8	3.3	2.6	2.8	0.5	0.9	Merksplas
	4.3 (0.5)	6.5 (2.2)	3.1 (0.9)	3.8 (1.4)	1.1 (0.5)	2.5 (1.0)	Chichibu
	11.8 (1.2)	12.0 (3.6)	10.9 (1.1)	9.1 (3.1)	1.0 (0.4)	3.1 (0.6)	Tanashi
Ca^2+^	11.1 (3.8)	15.5 (3.0)	8.6 (1.3)	9.2 (0.3)	3.3 (2.4)	7.3 (3.3)	Ontario
	8.1	8.9	6.9	5.7	1.2	3.4	Quebec
	− 0.5	− 3.1	1.6	− 0.3	− 2.1	− 2.2	Merksplas
	8.2 (2.1)	29.3 (4.9)	5.3 (2.4)	20.4 (4.1)	3.0 (1.3)	8.4 (2.0)	Chichibu
	20.8 (3.2)	28.6 (4.9)	16.1 (2.1)	21.1 (4.2)	4.7 (1.4)	7.4 (1.6)	Tanashi

Values in parentheses are standard deviations

The annual H^+^ SD value was significantly higher for coniferous than deciduous species at Chichibu and Tanashi (*p* < 0.001) (Table 2). At Chichibu and Tanashi, SF represented 78% and 83%, respectively, of the annual H^+^ SD value for coniferous species and 5% and 6%, respectively, for deciduous species (Table 2).

**Fig. 7:**
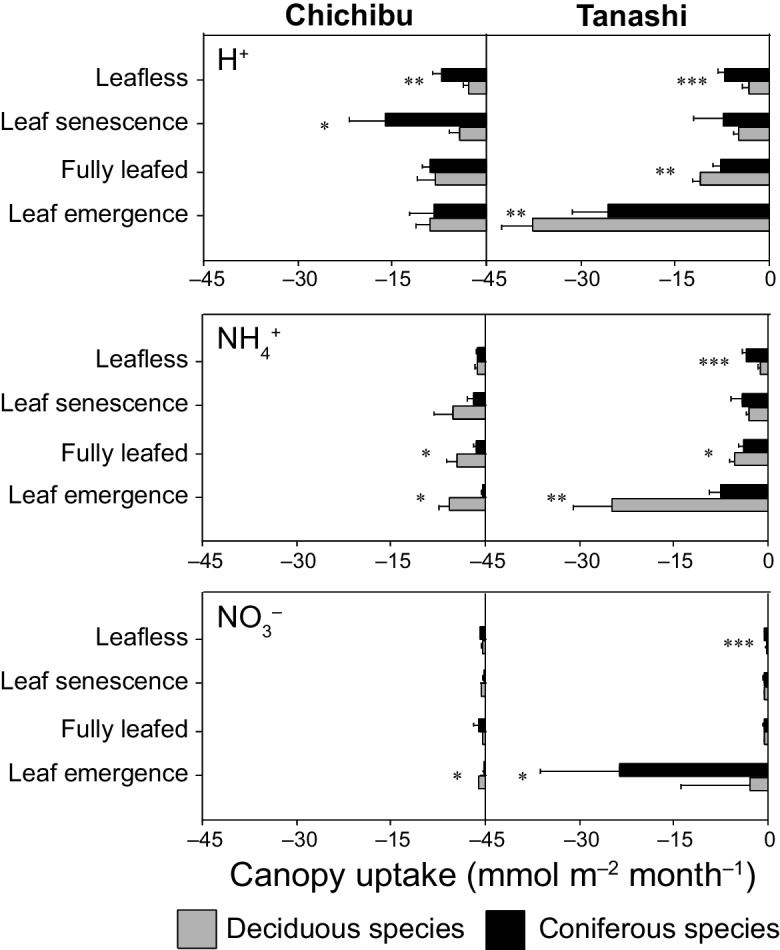
The 2-year average canopy uptake values (mmol m^−2^ month^−1^) of H^+^, NH_4_^+^, and NO_3_^−^ during each canopy phenological phase for deciduous species and coniferous species (*Cryptomeria japonica*) at Chichibu and Tanashi. The leaf emergence, fully leafed, leaf senescence, and leafless periods are defined in Fig. 3. * A paired *t*-test, *p* < 0.05, **t-test, *p* < 0.01, ***t-test, *p* < 0.001

The annual NH_4_^+^ SD value was significantly higher for deciduous than coniferous species at Chichibu (*p* < 0.01) (Table 2). By contrast, coniferous species showed significantly higher annual SD values of NH_4_^+^ (*p* < 0.01) and NO_3_^−^ (*p* < 0.001) than deciduous species at Tanashi (Table 2). At Chichibu, the annual NH_4_^+^ DD value was non-significantly higher than the CU value for deciduous species (*p* = 0.54) (Tables 3 and 5). At Tanashi, the annual DD values of NH_4_^+^ and NO_3_^−^ were significantly higher than the respective CU values for coniferous and deciduous species (*p* < 0.001 and *p* < 0.05, respectively), and the DD values were significantly higher for coniferous than deciduous species (*p* < 0.01 and *p* < 0.001, respectively) (Tables 3 and 5). The NH_4_^+^ CU value was significantly higher for deciduous than coniferous species in the leaf emergence and fully leafed periods at Chichibu and Tanashi (*p* < 0.05) (Fig. 7). The NH_4_^+^ DD value was significantly higher for deciduous than coniferous species in the leaf emergence, fully leafed, and leaf senescence periods at Chichibu (*p* < 0.05) (Fig. 5). By contrast, the NH_4_^+^ DD value was significantly higher for coniferous than deciduous species in the fully leafed and leafless periods at Tanashi (*p* < 0.01), while the NH_4_^+^ DD value was significantly higher for deciduous than coniferous species in the leaf emergence period (*p* < 0.01) (Fig. 5). In addition, at Chichibu, the NO_3_^−^ DD and CU values were significantly higher for deciduous than coniferous species in the leaf emergence period (*p* < 0.05) (Figs. 5 and 7). At Tanashi, the NO_3_^−^ DD value was higher for coniferous than deciduous species during all phenological periods except the leaf senescence period (*p* < 0.01) (Fig. 5).

**Table 5:** Comparison of the canopy uptake values (mmol m^−2^ year^−1^) of H^+^, NH_4_^+^, and NO_3_^−^ between deciduous (Dec) and coniferous (Con) species during the annual, growing, and dormant periods in this and previous studies

Canopy uptake (mmol m^–2^ year^–1^)							
Elements	Annual		Growing		Dormant		Site
	Dec	Con	Dec	Con	Dec	Con	
H^+^	55.9 (14.2)	61.0 (7.9)	43.9 (4.2)	41.0 (2.3)	13.0 (8.2)	22.4 (8.6)	Ontario
	57.9	49.4	52.0	35.5	4.9	14.3	Quebec
	6.1	1.0	3.8	0.8	0.3	0.5	Merksplas
	68.6 (15.7)	116.3 (19.4)	53.2 (29.4)	76.7 (13.4)	13.8 (7.6)	35.6 (8.3)	Chichibu
	121.4 (12.1)	106.7 (14.7)	108.1 (11.0)	78.0 (11.7)	13.4 (3.6)	28.3 (4.9)	Tanashi
NH_4_^+^	4.1 (1.8)	3.4 (0.8)	4.4 (1.9)	3.1 (0.8)	0.7 (0.5)	0.9 (0.3)	Ontario
	4.4	2.7	4.7	2.0	0.3	0.7	Quebec
	32.0	20.5	32.8	18.4	1.0	4.9	Merksplas
	36.3 (7.6)	17.7 (3.2)	31.2 (14.7)	10.7 (2.7)	5.8 (3.0)	6.4 (1.8)	Chichibu
	58.2 (7.0)	52.4 (6.4)	53.1 (6.7)	37.9 (5.4)	5.1 (1.7)	14.1 (2.2)	Tanashi
NO_3_^−^	1.4 (0.4)	1.7 (0.3)	1.0 (0.0)	1.1 (0.0)	0.3 (0.2)	0.6 (0.3)	Ontario
	1.4	1.2	1.1	0.8	0.1	0.3	Quebec
	4.7	2.1	5.6	2.0	1.1	0.5	Merksplas
	5.4 (1.2)	9.6 (1.3)	3.0 (1.1)	4.8 (1.5)	2.0 (1.1)	4.2 (1.7)	Chichibu
	6.9 (0.9)	9.3 (1.3)	5.9 (0.8)	7.0 (1.3)	0.9 (0.3)	2.2 (0.3)	Tanashi

Values in parentheses are standard deviations

## Discussion

### Sodium and chloride ions

At the low-atmospheric-deposition site (Chichibu), there were no differences in the annual Na^+^ DD or SD values between coniferous and deciduous species (Tables 2 and 3) because significant differences were observed only in the leaf senescence period (Fig. 5). Similarly, in Ontario, Canada, where the Na^+^ WD value was lower than that at Chichibu (Table 1), the annual SD value did not differ significantly between species (Neary & Gizyn, 1994) (Table 2) because the DD values did not differ between species in either the growing or dormant seasons (Table 3). In addition, at a low-atmospheric-deposition site in Quebec, Canada (Table 1), the Na^+^ SD and DD values were similar between coniferous and deciduous species (Houle et al., 1999) (Tables 2 and 3). These results suggest that differences in the annual SD values of Na^+^ between coniferous and deciduous species are minimally impacted by vegetation factors such as capture efficiency at low-deposition sites.

The annual Cl^−^ SD value was higher for coniferous than deciduous species at Chichibu (Table 2). This resulted from the significantly higher Cl^−^ DD value for coniferous than deciduous species during the leaf senescence and leafless periods (Fig. 5), which could be influenced by HCl gas concentrations at Chichibu. In Ontario and Quebec, where the Cl^−^ WD values were lower than that at Chichibu (Table 1), there were no significant differences in annual Cl^−^ SD values between species (Houle et al., 1999; Neary & Gizyn, 1994) (Table 2) because there were no differences in the DD values between species in the growing and dormant seasons (Table 3). Similar to Na^+^, the differences in annual Cl^−^ SD values between coniferous and deciduous species are minimally influenced by vegetation factors such as capture efficiency at low-deposition sites.

At the high-atmospheric-deposition site (Tanashi), the annual Na^+^ and Cl^−^ SD values were significantly higher for coniferous than deciduous species (Table 2) as a result of the higher DD values for the former species in the fully leafed and leafless periods (Fig. 5). In Merksplas, Belgium, where the Na^+^ WD value was higher than that at Tanashi (Table 1), the annual Na^+^ SD value was significantly higher for coniferous than deciduous species (De Schrijver et al., 2004) (Table 2) because the DD value was significantly higher for the former species in both the growing and dormant seasons (Table 3). Vegetation factors impacting DD include tree height (Griffith et al., 2015; Lovett & Reiners, 1986), LAI (Augusto et al., 2002), basal area (Zhang et al., 2022), vegetation type (evergreen vs. deciduous) (Erisman & Draaijers, 2003), and leaf shape (Woodcock, 1953). Tree height was higher for deciduous than coniferous species, and the difference in LAI was small between species at Tanashi and Quebec (Table 1). These results suggest that higher stand capture efficiency based on wide basal area and spiral leaf shape of coniferous canopies drive the differences in Na^+^ and Cl^−^ SD values between species in the growing (i.e., leafed) season. In the dormant season, the absence of leaves on deciduous species and differences in tree basal area drive the differences in the annual Na^+^ and Cl^−^ SD values between deciduous and coniferous species.

### Potassium, magnesium, and calcium ions

The annual K^+^ SD value was significantly higher for deciduous than coniferous species at Tanashi (Table 2) as a result of the low K^+^ DD value (Table 3) and increased K^+^ CL value for deciduous species in the leaf emergence and fully leafed periods (Fig. 6). Although not significant, the same trend was observed at Chichibu (Fig. 6, Tables 2 and 3). Similarly, three other studies found higher annual K^+^ SD values for deciduous than coniferous species because the K^+^ CL values were higher for deciduous than coniferous species in the growing season, even though these correlations were not significant (De Schrijver et al., 2004; Houle et al., 1999; Neary & Gizyn, 1994) (Tables 2 and 4). Similar to Chichibu and Tanashi, the previous studies also reported lower annual K^+^ DD than CL values (Tables 3 and 4). These trends indicate that differences in the annual K^+^ SD value between coniferous and deciduous species are influenced by the CL value of deciduous species in the growing season, especially the leaf emergence and fully leafed periods, even at sites with different atmospheric deposition values. In Japan, deciduous broad-leaved species generally have higher K^+^ levels in their leaves than *C. japonica* (Table 6). In addition, broad leaves are susceptible to the leaching of K^+^ (Rothe et al., 2002). The higher K^+^ CL value in deciduous than coniferous species in the leaf emergence and fully leafed periods was assumed to be due to differences in leaf K^+^ concentrations between deciduous and coniferous species. Therefore, differences in the K^+^ SD value between coniferous and deciduous species are influenced by the physiological state and leaf K^+^ concentration of tree species, regardless of the atmospheric deposition value.

**Table 6:** Average nutrient contents of leaves in Japan reported by previous studies

Family	Species	Location (Prefecture)	Nutrient content of leaves (%)			Reference
			K^+^	Mg^2+^	Ca^2+^	
Fagaceae	*Quercus serrata*	Kyoto	0.90	0.17	0.90	*Katagiri (1996)
	*Quercus crispula*		1.11	0.21	1.68	
	*Fagus crenata*		0.73	0.21	0.86	
	*Castanea crenata*	Shimane	0.93	0.34	0.85	†Katagiri (1977)
	*Quercus serrata*		0.66	0.37	1.46	
			0.87 (0.18)	0.26 (0.09)	1.15 (0.39)	
Cupressaceae	*Cryptomeria japonica*	Akita	0.48	0.21	1.39	‡Tsutsumi (1965)
		Nara	0.58	0.14	0.69	
			0.42	0.17	0.95	
			0.70	0.28	0.56	
			0.50	0.24	0.88	
			0.35	0.20	0.58	
			0.51 (0.12)	0.21 (0.05)	0.84 (0.05)	

Values in parentheses are standard deviations

^*^Katagiri, S. (1996). V. Material production and nutrient cycling in various forest ecosystems. In Iwatsubo, G (Ed.), *Modern Forestry  12 Forest ecology*, (pp. 189–293). Tokyo: Buneido Publishing Co., Ltd

^†^Katagiri, S. (1977). Studies on mineral cycling in a deciduous broad-leaved forest at Sanbe forest of Shimane university (IV) Concentration of nutrient elements of trees. *Bulletin of the Faculty of Agriculture, Shimane University*, *11*, 60–72

^‡^Tsutsumi, T. (1965). Amount of nutrients in trees of *Cryptomeria japonica*. *Transactions of the meeting of the Japanese Forestry Society*, *47*, 105–108

At Chichibu, the Mg^2+^ CL value was higher for deciduous species in the leaf emergence period and coniferous species in the leafless period; meanwhile, the Ca^2+^ CL was significantly higher for coniferous than deciduous species in the fully leafed and leafless periods (Fig. 6). Moreover, the annual Mg^2+^ SD value did not differ between tree species, and the Ca^2+^ SD value was significantly higher for coniferous than deciduous species (Table 2). The same trends were observed at low-deposition sites in Ontario and Quebec, Canada (Houle et al., 1999; Neary & Gizyn, 1994) (Table 2). This resulted from the higher Mg^2+^ CL value for deciduous species in the growing period and coniferous species in the dormant season (Table 4). In Ontario, the Ca^2+^ CL value was non-significantly higher for coniferous than deciduous species in the growing or dormant season; in Quebec, the Ca^2+^ CL value was significantly higher for coniferous species in the dormant season (Table 4). Leaf Mg^2+^ and Ca^2+^ concentrations did not differ between deciduous species and *C. japonica* (Table 6); however, broad leaves are more susceptible to leaching of Mg^2+^ than Ca^2+^ (Rothe et al., 2002). The lack of differences in annual Mg^2+^ SD values between coniferous and deciduous species resulted from differences in the Mg^2+^ CL values of each species between the growing and dormant seasons at low-deposition sites. By contrast, the higher annual Ca^2+^ SD value for coniferous than deciduous species resulted from higher CL from coniferous than deciduous species throughout the year. These results indicate that differences in annual Mg^2+^ and Ca^2+^ SD values between coniferous and deciduous species are influenced by the physiological characteristics of leaves at low-deposition sites.

The annual Mg^2+^ and Ca^2+^ DD values were > 20-fold lower than the respective CL values at the low-deposition site, Chichibu, but were only half the respective CL values at the high-deposition site (Tanashi; Tables 3 and 4). In addition, the Mg^2+^ and Ca^2+^ DD values were higher for coniferous than deciduous species during all phenological periods, except the leaf senescence period at Tanashi (Fig. 5). These trends indicate that Mg^2+^ and Ca^2+^ DD values also influence the differences in annual Mg^2+^ and Ca^2+^ SD values between coniferous and deciduous species at high-deposition sites. At a high-deposition site in Merksplas, Belgium (Table 1), the annual Mg^2+^ and Ca^2+^ SD values were higher for coniferous than deciduous species, although not significantly (De Schrijver et al., 2004) (Table 2); the Mg^2+^ and Ca^2+^ DD values were higher than the respective CL values and were also higher for coniferous than deciduous species throughout the year (Tables 3 and 4). These findings indicate that differences in annual Mg^2+^ and Ca^2+^ SD values between coniferous and deciduous species are affected by the CL and DD values at high-deposition sites. Mg^2+^ and Ca^2+^ CL and DD values between coniferous and deciduous species are influenced by the physiological characteristics of leaves and by stand capture efficiency, respectively.

### Hydrogen ion

The higher annual H^+^ SD for coniferous than deciduous species was the result of higher H^+^ SF for coniferous than deciduous species (Table 2). Generally, conifers supply high amounts of H^+^ via SF, due to the low pH of SF resulting from the comparatively higher dissolved organic concentrations of conifers (Inagaki et al., 1995; Parker, 1983; Thieme et al., 2019) and lower bark pH (Asplund et al., 2015) compared to deciduous species. Measurements in Ontario, Canada, revealed lower H^+^ SF values for conifers than in this study (Table 2) because the five most dominant species were selected for SF sampling in the former study (Table 1), which also found higher H^+^ SF levels for coniferous than deciduous species (Neary & Gizyn, 1994) (Table 2). Other studies conducted in Quebec, Canada, and Merksplas, Belgium, did not consider inputs of H^+^ from SF in the evaluation of H^+^ SD values, and the SD of conifers was not greater than that of deciduous species (De Schrijver et al., 2004; Houle et al., 1999) (Table 2). Overall, the findings indicate that differences in the annual H^+^ SD value between coniferous and deciduous species are caused by differences in H^+^ SF between species, regardless of atmospheric deposition values.

### Sulfur, ammonium, and nitrate ions

The CU of nitrogen is controlled by leaf physiological activities (e.g., stomatal opening and photosynthesis rates) (Krupa, 2003; Lovett et al., 1996; Parker, 1983) and passive diffusion processes from water film into leaves (Hansen, 1996; Lovett et al., 1996; Schaefer et al., 1988). In addition, some studies reported canopy nitrification by using a dual isotope approach (Guerrieri et al., 2015; Templer et al., 2015; Watanabe et al., 2016). However, CBM does not account for possible nitrogen transformations occurring in tree canopies by epiphytes and/or microbes associated with foliage (Guerrieri et al., 2021). Therefore, this chapter is just focused on leaf physiological activities and passive diffusion processes for CU of nitrogen.

Birch showed the highest uptake rates in the leaf developing stage by the ^15^NH_4_^+^-labelled test (Adriaenssens et al., 2011). In addition, Adriaenssens et al. (2012) reported a strong negative net TF flux for NO_3_^−^ in a beech canopy during the leaf development period, which was related to NO_3_^−^-N assimilation. At both Chichibu and Tanashi, we found that the NH_4_^+^ CU value was significantly higher for deciduous than coniferous species during the leaf emergence period (Fig. 7). This trend is considered the result of increased CU by leaf physiological activities of deciduous species in the leaf emergence period. In addition, water films on leaves, which form when particles are dry-deposited under relatively humid conditions, reduce cuticular resistance (Burkhardt & Eiden, 1994). Gaseous deposition of NH_3_, NO_y_, and SO_2_ from the atmosphere to the plant surface is increased in the presence of such films; as the gases dissolve into the water in the film, their concentrations rise, thereby enhancing passive diffusion from water film into the needle (Adriaenssens et al., 2012; Sase et al., 2008). De Schrijver et al. (2004) suggested that CU by deciduous species increases in the growing season when cuticles are thinner and wettability is greater. At the low-deposition site (Chichibu), we found that the SO_4_^2−^ and NH_4_^+^ DD values were significantly higher for deciduous than coniferous species in the fully leafed and leaf senescence periods (Fig. 5). The relative humidity during summer was higher at Chichibu than Tanashi because a large area around Chichibu is forested (Fig. 4). In addition, fog occurs frequently at Chichibu (Imamura et al., 2020b). Therefore, the increased SO_4_^2−^ and NH_4_^+^ DD values for deciduous species in the growing season are attributable to the result of the presence of water films on leaves. In addition, the NH_4_^+^ CU value for deciduous species also could be increased in the growing season via passive diffusion from water film into leaves (Fig. 7).

While the NH_4_^+^ CU value was significantly higher for deciduous than coniferous species in the leaf emergence period, the NH_4_^+^ DD value was significantly higher for deciduous than coniferous species during the growing season (Figs. 5 and 7). Therefore, the annual NH_4_^+^ SD value has been higher for deciduous than coniferous species because the annual NH_4_^+^ DD value was higher than the annual NH_4_^+^ CU value for deciduous species. The annual SO_4_^2−^ SD value did not differ between coniferous and deciduous species (Table 2); this was the result of a higher SO_4_^2−^ DD value for deciduous species in the fully leafed and leaf senescence periods and higher value for coniferous species in the leafless period (Fig. 5). At a low-deposition site in Quebec, Canada (Table 1), the significantly higher annual NH_4_^+^ SD value for deciduous than coniferous species resulted from a higher NH_4_^+^ DD value for the former species throughout the year (Houle et al., 1999) (Tables 2 and 3). Differences in the annual SO_4_^2−^ SD value between deciduous and coniferous species were influenced by leaf wettability, and those of NH_4_^+^ were influenced by leaf uptake (a phenological factor), as well as the DD and CU driven by leaf wettability and diffusion processes from water film into leaves at the low-deposition site.

The NO_3_^−^ CU value was higher for deciduous than coniferous species in the leaf emergence period at Chichibu (Fig. 7). However, there was no difference between species in the NO_3_^−^ DD value during other periods (Fig. 5). While the NH_4_^+^ DD value for deciduous species increased in fully leafed and leaf senescence periods (relatively humid conditions), the NO_3_^−^ DD value did not increase during same periods at Chichibu (Fig. 5). This is because water films had no impact on the diffusion processes, possibly due to low gas concentrations (NO: below the LOQ; NO_2_: 1.2 ppb). In Quebec, CU of NO_3_^−^ was reported in the leaf senescence period. In addition, the annual NO_3_^−^ SD value was significantly higher for deciduous than coniferous species because the NO_3_^−^ DD value was higher for the former species throughout the year (Houle et al., 1999) (Tables 2 and 3). The gas concentrations in 1999 in Quebec (NO: 2.3 ppb; NO_2_: 8.0 ppb; National Air Pollution Surveillance Program, 2022) were higher than those at Chichibu during the present study; thus, water film formation increased the DD of NO_3_^−^ for deciduous species. Overall, differences in the annual NO_3_^−^ SD value between deciduous and coniferous species were influenced by both phenological factors and diffusion processes, although atmospheric gas concentrations also impact diffusion.

At Tanashi, the significantly higher annual SO_4_^2−^ SD value for coniferous than deciduous species (Table 2) resulted from the higher SO_4_^2−^ DD values for coniferous than deciduous species in the fully leafed and leafless periods (Fig. 5). The NH_4_^+^ and NO_3_^−^ CU values were higher in the leaf emergence period (Fig. 5), and the annual NH_4_^+^ and NO_3_^−^ DD values were significantly higher than the respective CU values for both species (Tables 3 and 5). In addition, the NH_4_^+^ and NO_3_^−^ DD values were higher for coniferous than deciduous species in the fully leafed and leafless periods (Fig. 5). Therefore, the annual NH_4_^+^ and NO_3_^−^ SD values were significantly higher for coniferous than deciduous species (Table 2). In Ontario, Canada, where the SO_4_^2−^ WD value was higher than that at Tanashi (Table 1), the annual SO_4_^2−^ SD value was significantly higher for coniferous than deciduous species (Neary & Gizyn, 1994) (Table 2) because DD was significantly higher for coniferous than deciduous species in the growing season (Table 3). Similarly, the annual SO_4_^2−^ SD value was non-significantly higher for coniferous than deciduous species in Quebec (Houle et al., 1999) (Table 2) because the DD was higher for the former species in the growing and dormant seasons (Table 3). In Merksplas, Belgium, where the NH_4_^+^ and NO_3_^−^ WD values were higher than those in Tanashi (Table 1), the annual NH_4_^+^ and NO_3_^−^ DD values were higher than the respective CU values (Tables 3 and 5). In addition, the annual NH_4_^+^ and NO_3_^−^ DD and SD values were higher for coniferous than deciduous species (De Schrijver et al., 2004) (Tables 2 and 3). These results indicate that the annual SO_4_^2−^, NH_4_^+^, and NO_3_^−^ SD values are higher for coniferous than deciduous species as a result of increased DD (due to a higher capture efficiency) for coniferous species in the growing season, as well as the absence of leaves on deciduous species in the dormant season at high-deposition sites.

## Conclusion

This research explained the factors affecting the difference of dissolved ion inputs to the forest floor between coniferous and deciduous species by comparing seasonal variations of dry deposition and canopy exchange at two different atmospheric deposition conditions. Whereas this research is regional and based on limited observation data, this research cleared that the atmospheric deposition affected to vegetation factor, especially the capture efficiency of coniferous trees for Na^+^, Cl^−^, Mg^2+^, Ca^2+^, SO_4_^2−^, NH_4_^+^, and NO_3_^−^. In contrast, atmospheric deposition had no impact on canopy leaching of K^+^, Mg^2+^, and Ca^2+^ and neutralization between species. This suggests that information on atmospheric depositions in the study area could be important to estimate correctly different values of dissolved ion inputs to forest floor between deciduous and coniferous forests.

### Supplementary Information

Below is the link to the electronic supplementary material.

Supplementary file1 (XLSX 139 KB)

## Data Availability

Data is supplied by supplementary file.
