# Correction to: Factors influencing the difference in dissolved ion inputs to the forest floor between deciduous and coniferous stands: comparison under high and low atmospheric deposition conditions

**DOI:** 10.1007/s10661-024-12515-3

**Published:** 2024-03-12

**Authors:** Naohiro Imamura, Nobuhito Ohte, Nobuaki Tanaka

**Affiliations:** 1 Hokkaido Research Center, Forestry and Forest Products Research Institute, Toyohira‑Ku, Sapporo, Hokkaido Japan; 2 Graduate School of Informatics, Kyoto University, Sakyo‑Ku, Kyoto, Japan; 3 The University of Tokyo Hokkaido Forest, The University of Tokyo Forests, Graduate School of Agricultural and Life Sciences, The University of Tokyo, Furano, Hokkaido Japan

**Correction to: Environ Monit Assess (2024) 196:1**

10.1007/s10661-023-12132-6

The original version of this article unfortunately contained an error in the affiliation section.

The affiliation of the third author Nobuaki Tanaka is incorrect.

The third affiliation should be deleted from the affiliation section and fourth affiliation should be corrected from “The University of Tokyo Hokkaido Forest, The University of Tokyo Forests, Furano, Hokkaido, Japan” to “The University of Tokyo Hokkaido Forest, The University of Tokyo Forests, Graduate School of Agricultural and Life Sciences, The University of Tokyo, Furano, Hokkaido, Japan”

The corrected affiliation is shown in the affiliation section.

